# Molecular characteristics of tubo-ovarian carcinosarcoma at different anatomic locations

**DOI:** 10.1007/s00428-024-03821-9

**Published:** 2024-05-11

**Authors:** Ben Davidson, Arild Holth, Kristina Lindemann, Ane Gerda Zahl Eriksson, Thale Andrea Nilsen, Annette Torgunrud

**Affiliations:** 1 Department of Pathology, Norwegian Radium Hospital, Oslo University Hospital, Montebello, N-0310 Oslo, Norway; 2 Faculty of Medicine, University of Oslo, Institute of Clinical Medicine, N-0316 Oslo, Norway; 3 Department of Gynecologic Oncology, Norwegian Radium Hospital, Oslo University Hospital, Montebello, N-0310 Oslo, Norway; 4 Department of Tumor Biology, Norwegian Radium Hospital, Oslo University Hospital, Montebello, N-0310 Oslo, Norway

**Keywords:** Carcinosarcoma, Effusion, Solid metastasis, Targeted sequencing, TP53

## Abstract

Carcinosarcoma (CS) is an uncommon and clinically aggressive malignancy. The objective of the present study was to characterize the molecular features of CS at various anatomic locations, including serous effusions. Specimens (*n* = 32) consisted of 25 biopsies/surgical resection specimens and 7 serous effusions (6 peritoneal, 1 pleural) from 25 patients. Fresh-frozen cell pellets and surgical specimens underwent targeted next-generation sequencing covering 50 unique genes. A total of 31 mutations were found in 25 of the 32 tumors studied, of which 1 had 3 mutations, 4 had 2 different mutations, and 20 had a single mutation. The most common mutations were in *TP53* (*n* = 25 in 24 tumors; 1 tumor with 2 different mutations), with less common mutations found in *RB1* (*n* = 2), *MET* (*n* = 1), *KRAS* (*n* = 1), *PTEN* (*n* = 1), and *KIT* (*n* = 1). Patient-matched specimens harbored the same *TP53* mutation. Tumors with no detected mutations were more common in serous effusion specimens (3/7; 43%) compared with surgical specimens (4/25; 16%). In conclusion, the molecular landscape of CS is dominated by *TP53* mutations, reinforcing the observation that the majority of these tumors develop from high-grade serous carcinoma. Whether CS cells in serous effusions differ from their counterparts in solid lesions remains uncertain.

## Introduction

Carcinosarcoma (CS) is an uncommon and clinically aggressive cancer characterized morphologically by biphasic growth, with both epithelial and sarcomatous components present. The sarcomatous component may be homologous (spindle cell) or heterologous, the latter most commonly consisting of skeletal muscle or cartilage. CS is most commonly diagnosed in the uterine corpus, but may have origin in other gynecologic organs, including the adnexal region [1]. A recent study of 82 ovarian CS showed that 79% contained high-grade serous carcinoma (HGSC) elements, while the carcinomatous component was endometrioid in the remaining 21% cases. Patients with CS had significantly poorer response to chemotherapy and shorter survival compared with matched patients with HGSC [2].

Several studies analyzing the molecular characteristics of gynecologic CS have been published in recent years. The majority of these reports included both uterine and ovarian tumors, with the former constituting the majority of cases [3–7]; reviewed in [8], though two more recent studies have analyzed only ovarian CS [9, 10]. Of the latter, one study analyzed both ovarian and extra-ovarian tumors [9], whereas the most recent study focused on the ovarian tumors [10].

Solid CS metastases most frequently, though not exclusively, consist of the carcinomatous component of the tumor. Dissemination of this tumor to serous effusions (peritoneal, pleural, or pericardial), an anatomic site in which detection of metastatic sarcomas is exceedingly rare, is almost exclusively in the form of carcinoma [11]. Whether this special feature has any association with the molecular features of tumor cells remains unclear.

A previous study from our institution [12] analyzed 103 effusion specimens, of which the majority were HGSC. However, mutation analysis focused only on 6 genes (*TP53*, *PIK3CA*, *KRAS*, *HRAS*, *NRAS*, and *BRAF*), and only 1 of the 6 CS effusions analyzed carried *TP53* mutation, with no additional findings observed in these tumors. The frequent origin of CS in HGSC suggests that *TP53* mutation should be more common, and that other mutations related to the latter tumor may be present. This prompted us to study CS at different anatomic sites, including effusion specimens, using a different, and more comprehensive molecular platform.

## Material and methods

### Patients and specimens

The study material consisted of a series of 32 specimens, including 25 biopsies/surgical resections and 7 serous effusions (6 peritoneal, 1 pleural) from 25 patients, submitted to the Department of Pathology at the Norwegian Radium Hospital for routine diagnostic purposes during the period 2000–2021. One specimen was available from 21 patients, whereas 4 patients had tumors from several anatomic sites, including 2 patients with 2 tumors, 1 patient with 3 tumors, and 1 patient with 4 tumors.

The majority of CS develop from HGSC, the majority of which have origin in the fallopian tube. As the SEE-FIM (Sectioning and Extensively Examining the Fimbria) procedure, required for assessing whether the tumor originates from the fallopian tube, has not been applied prior to 2014 at our institution, and as the majority of cases predate this year, ovarian specimens are referred to as such, without reference to primary vs. metastatic location, though the latter is assumed for the majority.

Effusions were centrifuged immediately after tapping, and cell pellets were frozen at − 70 °C in equal amounts of RPMI 1640 medium (GIBCO-Invitrogen, Carlsbad, CA) containing 50% fetal calf serum (PAA Laboratories GmbH, Pasching, Austria) and 20% dimethylsulfoxide (Merck KGaA, Darmstadt, Germany). Cellblocks were prepared using the thrombin clot method. Surgical specimens were frozen at − 70 °C without any treatment.

Effusions and surgical specimens were reviewed and diagnosed based on the 2014/2020 WHO criteria by an experienced pathologist with sub-specialty in cytopathology and gynecologic pathology (BD), the former as part of a study of clinicopathologic parameters [13]. For effusions, diagnosis was based on morphology in Diff-Quik-stained and PAP-stained smears, H&E sections from cellblocks, and immunohistochemistry (IHC), the latter consisting of epithelial (PAX8, claudin-4, B72.3) and mesothelial (calretinin) markers. The pre-operative biopsy was additionally assessed in all cases with available material, and the surgical specimen was assessed in patients who received upfront surgery.

Tumor cell content was assessed in both effusions and surgical specimens, the latter by evaluation of frozen sections, with minimum set at 30%. The majority of specimens contained > 50% tumor cells. Frozen sections from the analyzed tissue were additionally assessed for the presence of carcinoma vs. sarcomatous elements.

Clinicopathologic data are presented in Table 1. Chemotherapy data were available for 23 patients, all of whom received platinum-based therapy at diagnosis. Chemoresponse was assessed in 22 patients, of whom 17 had complete response, 3 partial response, 1 stable disease, and 1 progression.

**Table 1:** Clinicopathologic parameters (*n* = 25 patients; 32 tumors)

Case	Specimen type	Anatomic site	Previous chemo	Age	CA 125	FIGO stage	Upfront therapy	RD	PFS	OS	Status
1	Effusion	Pleura	No	64	15,000	IV	Surgery	≤ 1	15	31	DOD
2	Effusion	Peritoneum	No	76	1588	III	Chemo	≤ 1	8	18	DOD
3	Effusion	Peritoneum	No	67	2600	III	Surgery	> 1	3	12	DOD
4	Effusion	Peritoneum	Yes	60	2223	IV	Chemo	≤ 1	0	6	DOD
5	Effusion	Peritoneum	No	74	717	III	Surgery	≤ 1	21	40	DOD
6	Effusion	Peritoneum	No	74	1096	III	Surgery	0	7	16	DOD
7	Effusion	Peritoneum	No	63	473	III	Surgery	> 1	29	31	DOD
8	Biopsy	Colon	Yes	72	87	II	Surgery	NA	15	32	DOD
9	Biopsy	Ovary	No	65	NA	III	NA	NA	NA	21	DOD
10	Biopsy	Ovary	No	60	NA	III	NA	NA	NA	16	DOD
11	Biopsy	Vagina	No	64	1650	IV	Surgery	NA	15	65	DOD
	Biopsy	Peritoneum									
12	Biopsy	Ovary	No	63	1123	III	Surgery	0	19	57	DOD
13	Biopsy	Ovary	Yes	73	1018	NA	Chemo	NA	43	53	DOD
14	Biopsy	Intestine	No	82	118	III	Chemo	NA	9	12	DOD
	Biopsy	Bladder									
	Biopsy	Peritoneum									
	Biopsy	Peritoneum									
15	Biopsy	Ovary	No	74	264	III	Surgery	0	4	17	DOD
16	Biopsy	Ovary	No	70	1095	III	Surgery	0	29	115	DOD
17	Biopsy	Ovary	No	58	64	II	Surgery	0	NA	144	DOD
18	Biopsy	Peritoneum	No	67	296	III	Surgery	0	NA	104	DOC^*a*^
19	Biopsy	Tube	No	70	269	III	Surgery	0	77	117	DOD
20	Biopsy	Ovary	No	60	176	I	Surgery	0	NA	120	DOD
21	Biopsy	Ovary	No	53	255	IV	Surgery	0	7	27	DOD
22	Biopsy	Ovary	Yes	66	1744	IV	Chemo	0	9	14	DOD
	Biopsy	Omentum									
23	Biopsy	Ovary	No	76	872	III	Chemo	0	12	99	DOD
24	Biopsy	Ovary	Yes	68	640	IV	Chemo	0	18	37	DOD
25	Biopsy	Ovary	No	62	804	IV	Surgery	0	27	54	DOD
	Biopsy	Omentum									
	Biopsy	Diaphragm									

Abbreviations: *RD* residual disease, *PFS* progression-free survival, *OS* overall survival, *NA* not available, *DOD* dead of disease, *DOC* dead of other cause

^a^Died of esophageal cancer

Informed consent was obtained according to national and institutional guidelines. Study approval was given by the Regional Committee for Medical Research Ethics in Norway (REK # S-04300).

### Molecular analysis

Effusions were thawed by diluting the cells 1:10 in PBS. The cell pellet was spun down at 1000 rpm for 10 min, and the supernatant was discarded. The cell pellet was then re-suspended in 200 µl of PBS. DNA from effusions and solid tumors were extracted using the NucleoSpin Tissue, Mini kit for DNA from cells and tissue (MACHEREY–NAGEL Düren*,* Germany).

DNA purity was measured using NanoDrop (Thermo Fisher Scientific, Waltham MA, USA). Median absorbance ratio 260/280 was 1.9 (1.8–2.1), and concentrations were determined with the Qubit fluorometer (Thermo Fisher Scientific). Targeted next-generation sequencing was performed with the Ion GeneStudio S5 system and was analyzed using the Ion AmpliSeq™ Cancer Hotspot Panel v2, covering 50 unique genes. The median coverage of called variants was 1991, enabling detection of variants down to 1% allele frequency. Variants were called, annotated, filtered with Ion Reporter Software V.5.10 (Thermo Fisher Scientific), and manually reassessed using Integrative Genomics Viewer.

This NGS assay does not include copy number variation (CNV) analysis.

### Statistical analysis

Statistical analysis was performed applying the SPSS-PC package (Version 29). Probability of < 0.05 was considered statistically significant.

For progression-free survival (PFS), follow-up time was calculated from the date of last chemotherapy treatment at diagnosis until the date of relapse, date of death from any cause, or end of follow-up. For overall survival (OS), follow-up time was calculated from the date of diagnosis until date of death from any cause or end of follow-up, whichever occurred first. Survival curves were plotted with the Kaplan–Meier method. The log-rank test was used to compare survival between the groups.

## Results

**Fig. 1:**
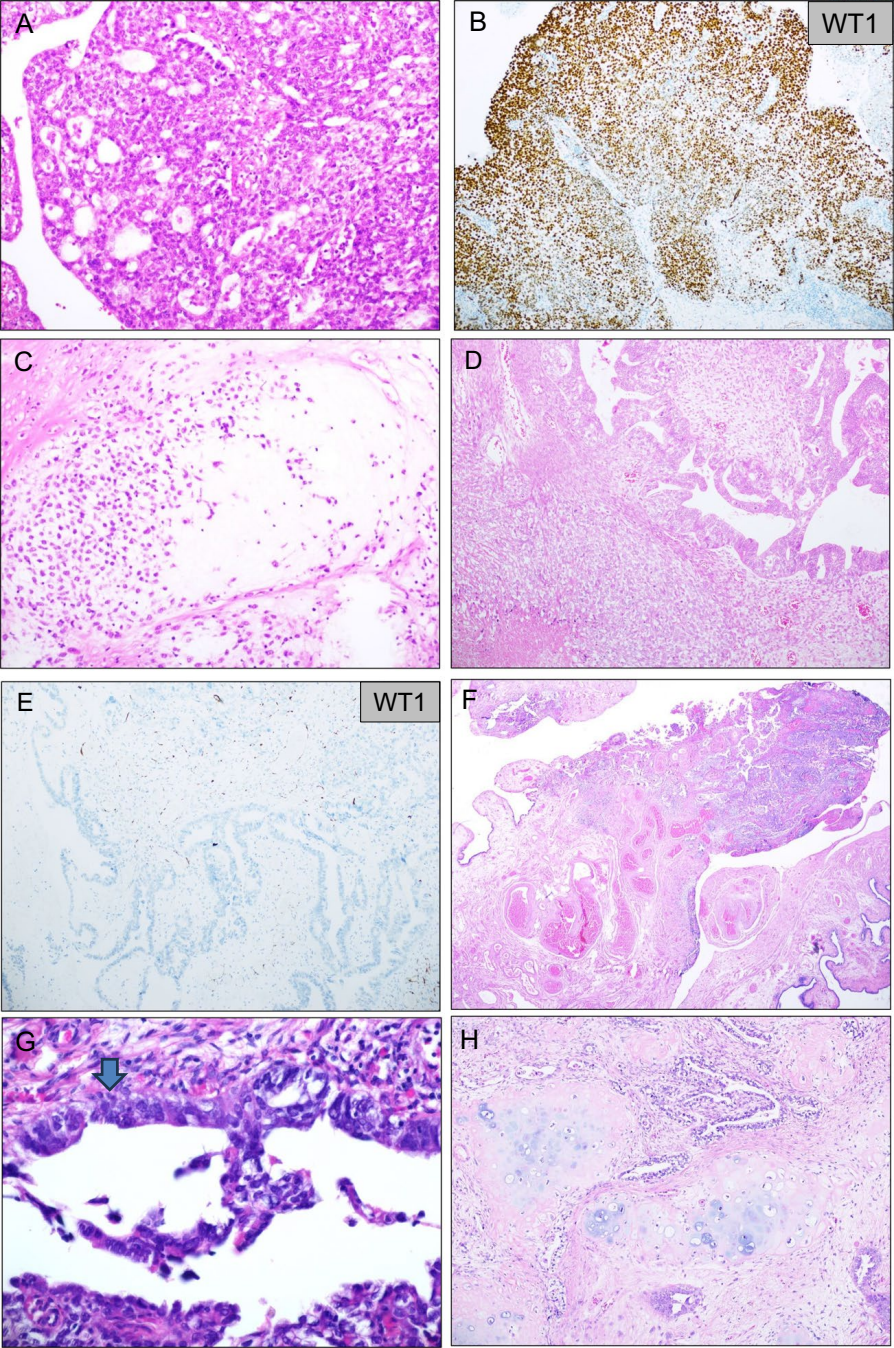
Carcinosarcoma (CS) histology and tumor origin. **A–C** Tumor with high-grade serous carcinoma (HGSC) morphological features in H&E stain (**A**), confirmed with WT1 immunostaining (**B**). Cartilage is evident in the sarcomatous component (**C**). **D–E** Tumor with endometrioid morphology in H&E stain (**D**), supported by negative WT1 immunostaining (**E**). **F–H** H&E-stained section from a primary fallopian tube tumor showing HGSC morphology (**F**), with a focus of serous tubal intraepithelial carcinoma (STIC; **G**). A sarcomatous element differentiated as cartilage is evident only in the ovarian metastasis (**H**)

With the exception of one tumor, the morphology of the epithelial component of the studied CS was of HGSC (Fig. 1A–C), the former tumor having endometrioid features and staining negative for WT1 (Fig. 1D, E).

Due to the abovementioned limitation with respect to the SEE-FIM protocol, tumor origin was not systematically assessed. However, tubal involvement, reinforcing the fact that the majority of these tumors are likely to arise from the fallopian tube, was observed in several cases, of which one had an unequivocal focus with serous tubal intraepithelial carcinoma (STIC; Fig. 1F–H).

Molecular data are summarized in Table 2. A total of 31 mutations were found in 25 of the 32 tumors studied, of which 1 had 3 mutations, 4 had 2 different mutations, and 20 had a single mutation. The most common mutations were in *TP53* (*n* = 25), with less common mutations found in *RB1* (*n* = 2), *MET* (*n* = 1), *KRAS* (*n* = 1), *PTEN* (*n* = 1), and *KIT* (*n* = 1). Patient-matched specimens from the 4 patients with multiple lesions carried the same *TP53* mutation. Tumors with no detected mutations were more common in serous effusion specimens (3/7; 43%) compared to surgical specimens (4/25; 16%), though the sample size was too small for comparative statistical analysis.

**Table 2:** Molecular findings

Case	Specimen type	Anatomic site	Histology^*a*^	Type	Gene	Function	Protein	Coding
1	Effusion	Pleura	Carcinoma	SNV	*TP53*	Missense	p.Tyr220Cys	c.659A > G
2	Effusion	Peritoneum	Carcinoma	SNV	*KIT*	Nonsense	p.Glu53Ter	c.157G > T
3	Effusion	Peritoneum	Carcinoma	SNV	*TP53*	Nonsense	p.Glu221Ter	c.661G > T
4	Effusion	Peritoneum	Carcinoma	No somatic variants				
5	Effusion	Peritoneum	Carcinoma	No somatic variants				
6	Effusion	Peritoneum	Carcinoma	No somatic variants				
7	Effusion	Peritoneum	Carcinoma	SNV	*KRAS*	Missense	p.Gly12Val	c.35G > T
				SNV	*TP53*	Missense	p.Arg248Gln	c.743G > A
8	Biopsy	Colon	Carcinoma	No somatic variants				
9	Biopsy	Ovary	Carcinoma	SNV	*RB1*	Missense	p.Arg455Pro	c.1364G > C
				SNV	*TP53*	Missense	p.Ser127Phe	c.380C > T
10	Biopsy	Ovary	Carcinoma	SNV	*TP53*	Nonsense	p.Arg306Ter	c.916C > T
11	Biopsy	Vagina	Carcinoma	SNV	*TP53*	Missense	p.Arg248Trp	c.742C > T
	Biopsy	Peritoneum	Carcinoma	SNV	*TP53*	Missense	p.Arg248Trp	c.742C > T
12	Biopsy	Ovary	Carcinoma + sarcoma	SNV	*TP53*	Missense	p.Arg267Pro	c.800G > C
13	Biopsy	Ovary	Carcinoma + sarcoma	No somatic variants				
14	Biopsy	Intestine	Carcinoma + sarcoma	SNV	*TP53*	Missense	p.Arg248Trp	c.742C > T
	Biopsy	Bladder	Carcinoma + sarcoma	SNV	*TP53*	Missense	p.Arg248Trp	c.742C > T
	Biopsy	Peritoneum	Carcinoma + sarcoma	SNV	*TP53*	Missense	p.Arg248Trp	c.742C > T
	Biopsy	Peritoneum	Carcinoma + sarcoma	SNV	*TP53*	Missense	p.Arg248Trp	c.742C > T
15	Biopsy	Ovary	Carcinoma	INDEL	*TP53*	Frameshift insertion	p.Asp186GlufsTer23	c.557_558insA
16	Biopsy	Ovary	Carcinoma	INDEL	*PTEN*	Non-frameshift deletion	p.Ser113_Glu114del	c.337_342del AGTG
				INDEL	*RB1*	Frameshift deletion	p.Asp145IlefsTer8	c.432delT
				SNV	*TP53*	Missense	p.Cys275Tyr	c.824G > A
17	Biopsy	Ovary	Sarcoma	INDEL	*TP53*	Frameshift deletion	p.Thr211PhefsTer4	c.631_632delAC
				SNV	*TP53*	Missense	p.Thr211Ala	c.631A > G
18	Biopsy	Peritoneum	Sarcoma	SNV	*TP53*	Missense	p.Phe134Leu	c.402 T > G
19	Biopsy	Tube	Carcinoma	SNV	*TP53*	Nonsense	p.Glu171Ter	c.511G > T
20	Biopsy	Ovary	Carcinoma + sarcoma	No somatic variants				
21	Biopsy	Ovary	Carcinoma + sarcoma	No somatic variants				
22	Biopsy	Ovary	Carcinoma	SNV	*TP53*	Missense	p.Cys135Trp	c.405C > G
	Biopsy	Omentum	Carcinoma	SNV	*TP53*	Missense	p.Cys135Trp	c.405C > G
23	Biopsy	Ovary	Carcinoma	SNV	*TP53*	Nonsense	p.Glu349Ter	c.1045G > T
24	Biopsy	Ovary	Carcinoma	SNV	*TP53*	Nonsense	p.Arg342Ter	c.1024C > T
				SNV	*MET*	Missense	p.Val176Met	c. 526G > A
25	Biopsy	Ovary	Carcinoma	SNV	*TP53*	Missense	p.Val173Leu	c.517G > T
	Biopsy	Omentum	Carcinoma	SNV	*TP53*	Missense	p.Val173Leu	c.517G > T
	Biopsy	Diaphragm	Carcinoma	SNV	*TP53*	Missense	p.Val173Leu	c.517G > T

^*a*^Histology = tumor component present in the frozen material analyzed in the present study

**Fig. 2:**
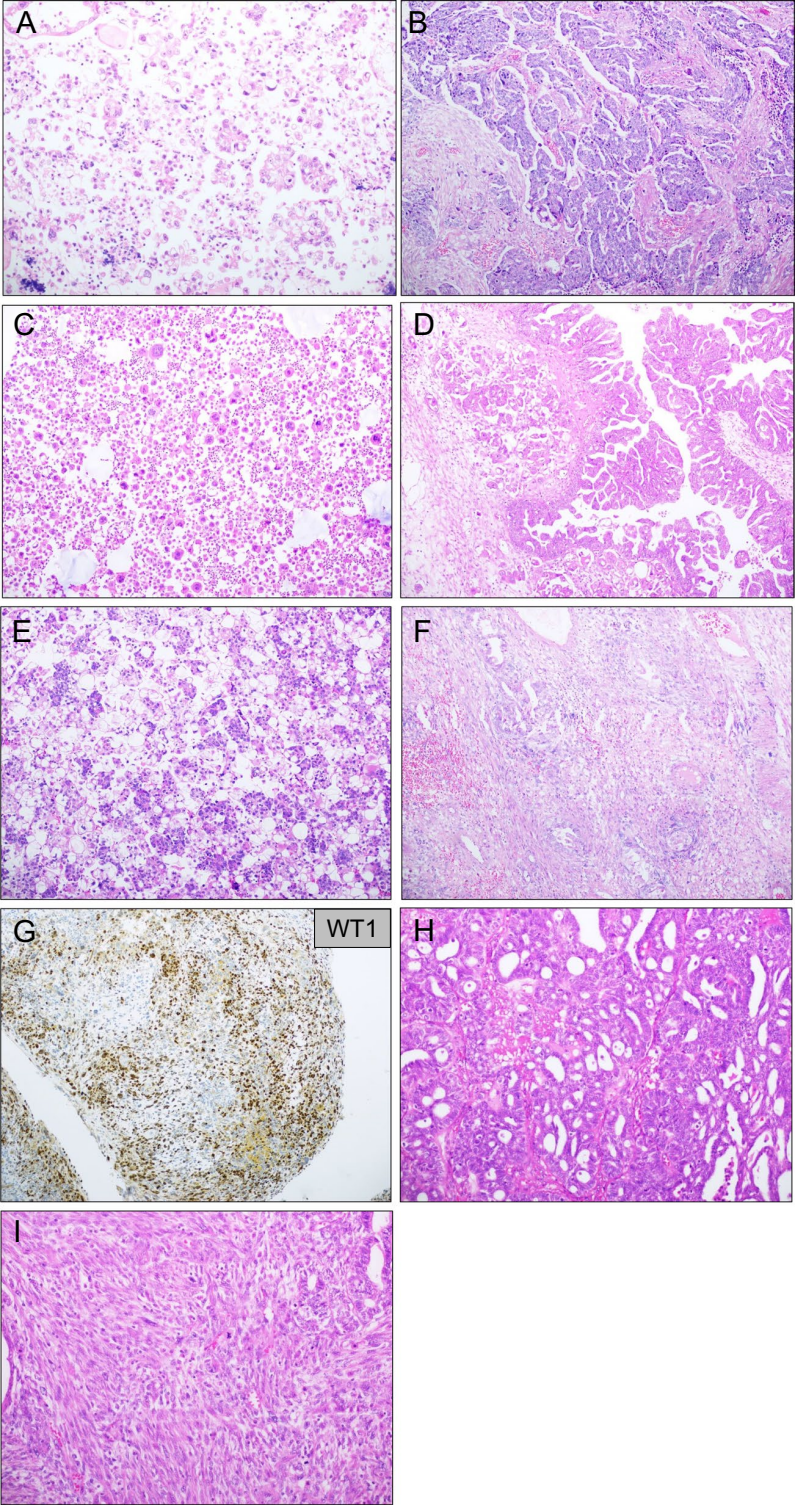
Tumors with less frequent mutation profile. **A–B** Tumor lacking *TP53* or other mutation. Morphology is equivocal in the effusion specimen (**A**; both HGSC and clear cell carcinoma may be relevant diagnoses), but with HGSC morphology in the ovarian specimen (**B**; both H&E stain). **C–D** Tumor with both *TP53* and *KRAS* mutation. Pleomorphic, predominantly dissociated tumor cells are seen in the effusion specimen (**C**), but the ovarian tumor showed typical HGSC morphology (**D**; both H&E stain). **E–G** Effusion (**E**) and ovarian tumor (**F**), both with characteristic HGSC morphology, from a tumor harboring only *KIT* mutation (both H&E stain). WT1 immunostain of the ovarian tumor is predominantly positive (**G**), supporting the morphological findings. **H–I** Tumor harboring *TP53*, *RB1*, and *PTEN* mutation, with HGSC morphology in the epithelial component (**H**), spindle cell morphology with no heterologous elements in the sarcomatous component (**I**; both H&E stain)

As specified above, tumors negative for all mutations included in the gene panel applied had features of HGSC in the epithelial tumor component (Fig. 2A and B). The same was true for the tumor harboring both *TP53* and *KRAS* mutation (Fig. 2C, D); the tumor harboring only *KIT* mutation (Fig. 2E–G); and the tumor harboring *TP53*, *RB1*, and *PTEN* mutation (Fig. 2H, I).

The tumor with endometrioid morphology in the epithelial component had no molecular alterations.

**Fig. 3:**
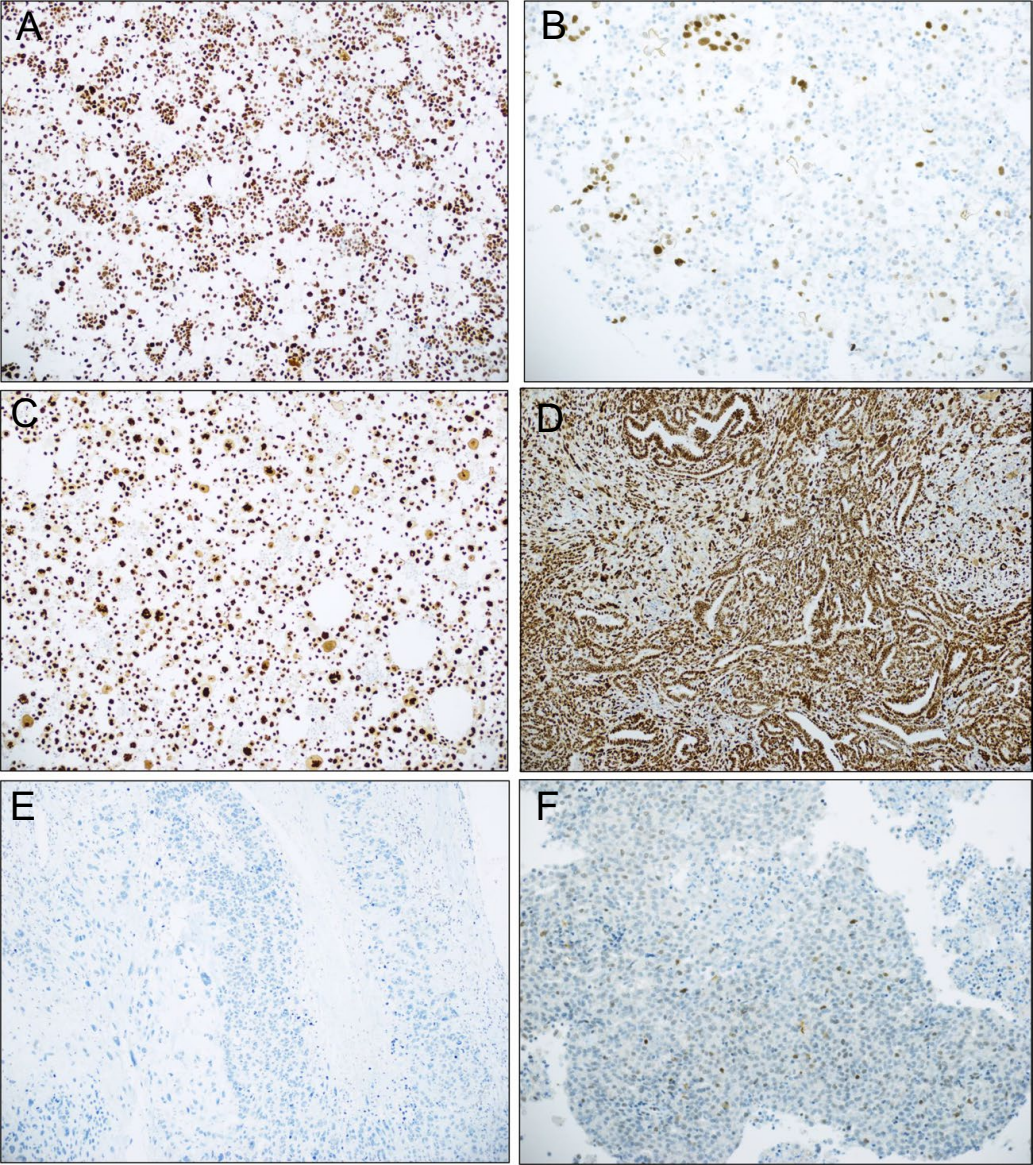
p53 immunostaining. **A** Effusion specimen (#2 in Table 2) with aberrant (diffusely positive) p53 expression. This tumor harbored *KIT* mutation, but no *TP53* mutation was found. **B** Effusion specimen (#4 in Table 2) with wild-type p53 staining pattern. No *TP53* mutation was found by NGS. **C** Effusion specimen (#7 in Table 2) with aberrant (diffusely positive) p53 expression. This tumor harbored *TP53* mutation. **D–E** Two surgical specimens with aberrant (diffusely positive and entirely negative) p53 staining pattern (#16 and 17 in Table 2). Both harbored *TP53* mutation. **F** Surgical specimen with wild-type p53 staining pattern (#20 in Table 2). No *TP53* mutation was found

In order to further investigate p53 status in the studied material, tumors were immunostained for p53 protein using a recently described protocol [14]. As patient-matched tumors carried identical *TP53* mutations, only one lesion from each patient was stained in the 4 cases with > 1 specimen. Nineteen tumors had aberrant staining pattern (15 diffusely positive, 3 with negative/null pattern, 1 with combined diffusely positive and cytoplasmic pattern) and 6 tumors stained with wild-type pattern (Fig. 3). Among tumors with wild-type p53 by IHC, 3 had no *TP53* mutations and 3 harbored mutations. Among the 19 tumors with aberrant staining pattern, NGS identified *TP53* mutations in 14 cases.

With the exception of one patient who died of other cancer, all patients in this study died of CS. For these 24 patients, PFS ranged from 0 to 77 months (mean = 18, median = 15), whereas OS ranged from 6 to 144 months (mean = 48, median = 31). The number of patients was deemed insufficient for robust analysis. However, an attempt was nevertheless made to see whether tumors with no mutations were associated with markedly different survival than their mutated counterparts. No significant differences were found between these two groups (*p* = 0.614 for OS; *p* = 0.663 for PFS; data not shown).

## Discussion

Adnexal CS have been consistently referred to as “ovarian” in previous publications, but their frequent origin in HGSC suggests that many are in fact of tubal origin, a fact which was clearly evident in the present study despite inadequate sampling of the fallopian tube. The first author of this study has seen several tumors with the morphology of HGSC in the fallopian tube that acquired the sarcomatous component in the ovarian metastasis, as was the case in the tumor shown in Fig. 1F–1H in the present study. Given this, these tumors should be referred to as tubo-ovarian rather than ovarian, in the same manner that adnexal carcinomas are. Finally, the predominance of HGSC as the epithelial component of adnexal CS is well in agreement with previous studies [2, 9]. It is also in agreement with recent reports assigning the majority of uterine CS to the copy number high/TP53 mutated group using the TCGA classification algorithm [15, 16].

With a postulated origin in the fallopian tube for the majority of tumors in our study, they should be perceived to consist predominantly of metastatic CS, as is likely the case with many of the ovarian tumors studied by other groups. Studies of extra-adnexal metastases from adnexal CS are nevertheless to date limited to a single study of surgical specimens [9], in which 18 ovarian and 12 extra-ovarian tumors were studied, and to a previous study of serous effusions from our hospital [12]. Given the high metastatic propensity and clinical aggressiveness of this cancer, the need to expand our knowledge in this area is evident.

As expected in a tumor originating primarily from HGSC, *TP53* was the most commonly mutated gene, and similar *TP53* mutation was found in patient-matched specimens from different anatomic locations, attesting to the central role of this molecular event in the biology of CS. This is in agreement with the previous above-mentioned of adnexal CS [9, 10], though the percentage of tumors with no detectable *TP53* mutation is somewhat higher in the present series. In this relatively small series, mutations were more common in surgical specimens compared to effusions. *TP53* mutations were nevertheless found in 3/7 effusions compared to 1/6 in the previous study of serous effusions, possibly owing to technical differences in the assays used. Technical factors need also be considered with respect to tumors with unequivocal HGSC morphology in the epithelial component that did not harbor mutation in this gene. Nevertheless, differences in p53 protein content among HGSC with different *TP53* mutations have been observed in analysis of public databases, including the TCGA cohort [17]. In another study of HGSC, discordant results were observed in analysis of *TP53* mutation status and p53 protein expression by IHC [18]. Finally, tumors with the morphological features of HGSC that have wild-type p53 staining pattern and/or lack *TP53* mutations have been described [19]. This suggests that both gene and protein status should be analyzed in selected cases, as recently recommended for adnexal HGSC [20].

Other genetic changes were uncommon in this series. The finding of infrequent mutations in *RB1*, *KRAS*, and *PTEN* is well in agreement with data from the Ho series, in which *KRAS* mutation and amplification was found in 4/18 tumors, *RB1* deletion in 2/18 tumors, and *PTEN* mutation in 2/18 cases tumors [9]. Mutations in these genes have also been reported in studies combining uterine and adnexal tumors [3, 4, 6].

We additionally found mutations in *MET* and *KIT* in 1 case each, which to the best of our knowledge have not been reported in tubo-ovarian CS, the former in a case harboring *TP53* mutation, the latter as single finding. *MET* mutation was found in 2/76 tumors in a study of endometrial CS, whereas *KIT* and/or *PDGFRA* mutations were found in 5 tumors in the same study [21], and our findings are thus comparable in terms of frequency.

Assessment of the prognostic role of the detected mutations in our series is limited by its size. We nevertheless did not observe any difference between women diagnosed with tumors harboring mutation compared to those with no detected ones. The same was true upon looking specifically at long-term survivals with OS > 100 months, or those with very poor survival (OS ≤ 24 months). Conclusive assessment of this issue requires larger studies.

In conclusion, analysis of mutation profiles in CS at different anatomic locations, the majority metastatic, shows predominance of *TP53* mutations, in agreement with the frequent detection of HGSC histology in the epithelial component of this tumor. We additionally report novel mutations in *MET* and *KIT* in tubo-ovarian CS. Whether metastasis to the serosal cavities in the form of effusion selects for non-mutated tumors awaits larger studies, though this does not appear to be associated with better outcome.

## Data Availability

Data is avaialable upon reasonable request. Queries should be addressed to one of the corresponding authors.
